# Arbaclofen in fragile X syndrome: results of phase 3 trials

**DOI:** 10.1186/s11689-016-9181-6

**Published:** 2017-06-12

**Authors:** Elizabeth Berry-Kravis, Randi Hagerman, Jeannie Visootsak, Dejan Budimirovic, Walter E. Kaufmann, Maryann Cherubini, Peter Zarevics, Karen Walton-Bowen, Paul Wang, Mark F. Bear, Randall L. Carpenter

**Affiliations:** 1 Departments of Pediatrics, Neurological Sciences, Biochemistry, Rush University Medical Center, 1725 West Harrison, Suite 718, Chicago, IL 60612 USA; 2 MIND Institute and Department of Pediatrics, University of California Davis Medical Center, 2825 50th Street, Sacramento, CA 95817 USA; 3 Department of Human Genetics, Emory University, 2165 N. Decatur Road, Decatur, GA 30033 USA; 4 Departments of Psychiatry &Behavioral Sciences, Kennedy Krieger Institute, the Johns Hopkins Medical Institutions, 716 N. Broadway, Room 246, Baltimore, MD 21205 USA; 5 Department of Neurology, Boston Children’s Hospital, Boston, MA 02115 and Greenwood Genetic Center, Greenwood, SC 29646, USA; 6 Seaside Therapeutics Inc, 124 Washington Street, Suite 101, Foxboro, MA 02035, USA; 7 Simons Foundation Autism Research Initiative, 160 Fifth Avenue, 7th Floor, New York, NY 10010, USA; 8 Autism Speaks, 1 East 33rd Street, 4th Floor, New York, NY 10016, USA; 9 The Picower Institute for Learning and Memory, Massachusetts Institute of Technology, 43 Vassar Street, 46-3301, Cambridge, MA 02139, USA; 10 Rett Syndrome Research Trust, 67 Under Cliff Rd, Trumbull, CT 06611, USA

**Keywords:** Fragile X syndrome, Arbaclofen, GABA agonist, FMR1, Targeted treatment, Neurodevelopmental disorder

## Abstract

**Background:**

Arbaclofen improved multiple abnormal phenotypes in animal models of fragile X syndrome (FXS) and showed promising results in a phase 2 clinical study. The objective of the study is to determine safety and efficacy of arbaclofen for social avoidance in FXS.

**Methods:**

Two phase 3 placebo-controlled trials were conducted, a flexible dose trial in subjects age 12–50 (209FX301, adolescent/adult study) and a fixed dose trial in subjects age 5–11 (209FX302, child study). The primary endpoint for both trials was the Social Avoidance subscale of the Aberrant Behavior Checklist-Community Edition, FXS-specific (ABC-C_FX_). Secondary outcomes included other ABC-C_FX_ subscale scores, Clinical Global Impression-Improvement (CGI-I), Clinical Global Impression-Severity (CGI-S), and Vineland Adaptive Behavior Scales, Second Edition (Vineland-II) Socialization domain score.

**Results:**

A total 119 of 125 randomized subjects completed the adolescent/adult study (*n* = 57 arbaclofen, 62 placebo) and 159/172 completed the child study (arbaclofen 5 BID *n* = 38; 10 BID *n* = 39; 10 TID *n* = 38; placebo *n* = 44). There were no serious adverse events (AEs); the most common AEs included somatic (headache, vomiting, nausea), neurobehavioral (irritability/agitation, anxiety, hyperactivity), decreased appetite, and infectious conditions, many of which were also common on placebo. In the combined studies, there were 13 discontinuations (*n* = 12 arbaclofen, 1 placebo) due to AEs (all neurobehavioral). The adolescent/adult study did not show benefit for arbaclofen over placebo for any measure. In the child study, the highest dose group showed benefit over placebo on the ABC-C_FX_ Irritability subscale (*p* = 0.03) and Parenting Stress Index (PSI, *p* = 0.03) and trends toward benefit on the ABC-C_FX_ Social Avoidance and Hyperactivity subscales (both *p* < 0.1) and CGI-I (*p* = 0.119). Effect size in the highest dose group was similar to effect sizes for FDA-approved serotonin reuptake inhibitors (SSRIs).

**Conclusions:**

Arbaclofen did not meet the primary outcome of improved social avoidance in FXS in either study. Data from secondary measures in the child study suggests younger patients may derive benefit, but additional studies with a larger cohort on higher doses would be required to confirm this finding. The reported studies illustrate the challenges but represent a significant step forward in translating targeted treatments from preclinical models to clinical trials in humans with FXS.

## Background

Fragile X syndrome (FXS) is an X-linked condition that, with a prevalence estimated at 1 in 4000 males and 1 in 8000 females [1], is the most common known inherited cause of intellectual disability and the most common single gene cause of autism spectrum disorder (ASD). FXS is associated with characteristic behavioral features such as attention deficits, hyperactivity, anxiety, mood lability, and in some cases aggression, as well as autistic features including prominent perseverative behavior and social impairments [2]. At present, no symptomatic or disease-modifying treatments for FXS have received regulatory approval.

FXS is typically caused by an expansion (>200) of a CGG trinucleotide repeat sequence in the promoter region of the *Fragile X Mental Retardation 1* (*FMR1*) gene. This expansion is associated with complete or partial methylation of the *FMR1* promoter, resulting in loss or significant reduction of expression of the gene product, FMRP (fragile X mental retardation protein) [3]. Females with FXS tend to be less severely affected than males due to expression of the normal X chromosome [4]. FMRP is an RNA-binding protein that modulates the dendritic localization and translation of several hundred mRNA ligands [5]. In the *Fmr1* knockout mouse, the absence of FMRP leads to excessive protein synthesis downstream of signaling pathways coupled to group I metabotropic glutamate receptors (mGluRs), in particular mGluR5. Inhibition of mGluR5 and downstream signaling has been shown to correct a wide array of disease phenotypes in animal models of FXS [6].

Glutamatergic transmission is under tight regulation by GABAergic inhibition, and deficiencies in *γ*-aminobutyric acid (GABA-mediated inhibitory neurotransmission have been identified in the hippocampus, striatum, somatosensory cortex, and amygdala of *Fmr1* knockout mice [7]. Use of GABA agonists has been suggested as a therapeutic strategy for FXS [8]. In fact, preclinical animal data showed rescue of glutamate-induced lethality, neuropathology, excessive protein translation, and abnormal courtship behavior in the *dfmr* mutant fly with GABAergic compounds [9]. In *Fmr1* knockout mice, studies have shown improvement in elevated plus maze performance with the GABA-A agonist alphaxalone, and protection from audiogenic seizures with the GABA-A agonist ganaxolone and the racemic GABA-B agonist baclofen [10]. In addition to ameliorating audiogenic seizures, treatment of *Fmr1* knockout mice with the (*R*)-(+)-enantiomer of baclofen corrects some core features of FXS pathophysiology, including excessive basal protein synthesis, increased activity of the mammalian target of rapamycin pathway, and abnormal spine density [11, 12].

Arbaclofen (STX209, R-baclofen) is a potent and selective agonist of GABA-B receptors and contrasts with (*S*)-(-)-baclofen with respect to metabolism, CNS transport, and activity [11]. In humans, arbaclofen shows pharmacokinetic properties similar to those of racemic baclofen, with high bioavailability and a terminal half-life of 4 to 5 h. Arbaclofen undergoes renal elimination with no significant metabolism [13]. Based on preclinical work, anecdotal clinical experience suggesting behavioral benefits from racemic baclofen in FXS and autism spectrum disorder (ASD), and data from transcranial magnetic stimulation (TMS) studies demonstrating enhancement of cortical inhibition by racemic baclofen [14], a phase 2 double-blind placebo-controlled flexible dose crossover trial of arbaclofen was conducted in (*n* = 63) subjects with FXS [15]. All subjects met severity criteria on the Aberrant Behavior Checklist-Community Edition (ABC-C) Irritability subscale, which was the primary endpoint based on FDA precedent for use of this scale for prior approval of risperidone and aripiprazole for irritability in ASD.

In that trial, arbaclofen showed no significant safety issues. Although there was no benefit for arbaclofen over placebo on the primary outcome, significant improvement over placebo was seen on a visual analog scale (VAS) for the three most severe parent-nominated behaviors and on the ABC-C_FX_ (ABC-C refactored for FXS population [16]) Social Avoidance subscale, with a trend in favor of arbaclofen also for blinded treatment preference as reported by clinicians and parents, the Clinical Global Impression-Severity (CGI-S), and Clinical Global Impression-Improvement (CGI-I). In a post hoc analysis, arbaclofen showed significant improvement over placebo in the more socially impaired subgroup (*n* = 27) for the treatment period preference (both clinician and parent), CGI-I, CGI-S, Vineland Adaptive Behavior Scales, Second Edition (Vineland-II) Socialization domain, ABC-C Lethargy/Social Withdrawal subscale, ABC-C_FX_ Social Avoidance subscale, and a responder analysis (CGI-I of much or very much improved and at least 25% improvement on ABC-C Lethargy/Social Withdrawal). The results were also more robust among subjects who met Diagnostic and Statistical Manual of Mental Disorders-Fourth Edition (DSM-IV) and Autism Diagnostic Interview-Revised (ADI-R) criteria for autistic disorder. Significantly more subjects were responders on the CGI-I scale when receiving arbaclofen vs. placebo (35 vs 18% overall; 50 vs. 6% autism) in the autism subgroup, although again the ABC-C Irritability was not sensitive to these effects. A majority of subjects enrolled in an open-label extension study, and some were able to be withdrawn from their concomitant medications, including from antipsychotics.

Based on these encouraging results from the phase 2 trial, two phase 3 placebo-controlled trials of arbaclofen, focused on treatment of social avoidance, were done in adolescents and adults (age 12–50) and in children (age 5–11) with FXS. The decision to conduct simultaneously two parallel studies was based upon a sense of urgency to attempt to obtain FDA approval and make arbaclofen available as rapidly as possible for the entire age range of individuals with FXS. Here, we report the results of both phase 3 trials.

## Methods

### Participants

Participants were males and females with a DNA-confirmed *FMR1* full mutation aged 12 to 50 years (adolescent/adult study), and aged 5–11 (child study). The age ranges are those preferred by FDA for developmental disorders, with the 5–11 age group representing predominantly prepubertal children with FXS and the 12–50-year-old group representing adolescents and adults with FXS for whom behavioral issues are overall very similar throughout the age range. Up to three concomitant psychoactive medications (including antiepileptic drugs), which were FDA-approved for the condition or symptom being treated, were permitted, but use of vigabatrin, tiagabine, riluzole, racemic baclofen was prohibited because of their GABAergic mechanisms. Also, participants could not be taking medications with anxiolytic properties (including serotonin reuptake inhibitors (SSRIs), tricyclic antidepressants, venlafaxine, buspirone, benzodiazepines that were administered on a regular daily schedule, and propranolol). It was not considered feasible in FXS, a rare condition with severe behavioral dysfunction, to enroll a cohort in the specified age ranges with no psychoactive medication treatment that would be sufficient size for appropriate power to detect drug effect. Further, allowance of standard-of-care background therapy would allow identification of effects contributed by arbaclofen which supersede those obtained from standard care. Pharmacological treatment regimens were required to be stable for 4 weeks and educational, behavioral, and other treatments stable for 2 months, prior to screening and for the duration of the study. Subjects with any previous seizure were required to be on anticonvulsant medication and seizure-free for 6 months or seizure-free for 3 years off of anticonvulsants. A score of 8 or greater on the parent-rated ABC-C Lethargy/Social Withdrawal subscale was required at the screening visit and visit 1 at the beginning of the treatment period. This cutoff was used because it was the median value observed in the prior phase 2 arbaclofen trial in FXS, and also defined in the trial post hoc analyses the group that demonstrated significant improvement on numerous measures including the ABC-C_FX_ Social Avoidance subscale. Caregivers watched a training video explaining how to rate the ABC-C before performing the rating at screening. Female subjects of childbearing potential were tested and excluded if they were pregnant. Female patients were required to follow an acceptable method of birth control throughout the study. The major exclusion criteria included, but were not limited to, impairment of renal function, evidence or history of malignancy, or any significant hematological, endocrine, cardiovascular, respiratory, hepatic, or gastrointestinal disease, and illicit drug use or alcohol abuse. Informed consent was obtained from the participant or a legal guardian or legally acceptable representative in all cases, and participants were enrolled if they met all inclusion criteria. The studies (clinicaltrials.gov identifiers NCT01282268 for adolescent/adult study, NCT01325220 for child study) were approved by the Institutional Review Boards governing each site.

### Study design

**Fig. 1:**
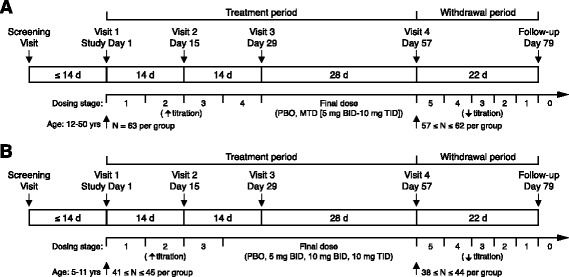
Design of adult/adolescent (**a**) and child (**b**) phase III arbaclofen studies

The studies were phase 3 randomized, double-blind, placebo-controlled, multisite, parallel group trials in adolescent/adults (209FX301, NCT01282268) and children (209FX302, NCT01325220) with FXS, conducted at 23 sites between May 2011 and December 2012 (adolescent/adult study) and 25 sites between June 2011 and June 2013 (child study) in the USA (Fig. 1 shows design of both studies). Study design complied with FDA GCP requirements and followed the standard elements in the CONSORT checklist guidelines. In the adolescent/adult study, drug was flexibly titrated every 7 days, starting at 5 mg BID, and then 10 mg BID, 10 mg TID, and 15 mg TID, until the maximal tolerated dose was established. In the child study, participants were assigned in a ratio of 1:1:1:1 to one of the following four fixed dose treatment arms: arbaclofen 5 mg BID, 10 mg BID, 10 mg TID, or placebo. Dosing was chosen based on FDA’s requirement that three parallel dose groups be enrolled in a placebo-controlled trial (fourth dose group receiving placebo). In addition, the dose that demonstrated efficacy in the phase 2 trial post hoc analyses was chosen as the middle dose and doses 50% lower and 50% higher were selected for the other two dose groups. Participants allocated to an arbaclofen arm-initiated therapy with 5 mg daily, and the dose was up-titrated every 7 days in steps (5 mg BID, 10 mg BID, then 10 mg TID) until the target dose was reached. Down-titration for dose adjustment was not allowed due to FDA’s preference for the most stringent assessment of tolerability; patients unable to tolerate their assigned dose were discontinued. Randomization was stratified based upon the use of antipsychotic medication. The total length of the double-blind treatment period was 8 weeks for both studies, including up-titration and then stable dosing for at least the final 4 weeks at the MTD (adolescent/adult study) or assigned fixed dose (child study).

Subjects returned for evaluations 2, 4, and 8 weeks after initiating double-blind treatment. After the 8-week treatment period, participants entered a withdrawal period, during which study drug was tapered weekly until off, according to the reverse of the up-titration schedules noted above, over 0–3 weeks (adolescent/adult study) or 1–3 weeks (child study). Phone calls occurred every 3–4 days during the first 29 days after randomization, when the drug was being titrated upwards; then every 2 weeks, during the stable dosing period; and then every 4–6 days, during down-titration at the end of the placebo-controlled dosing period. Participants returned for a close-out visit within 3 days of the last dose of study medication (adult/adolescent study) or at 11 weeks when off study medication (child study). Subjects who completed the 8-week double-blind treatment period or who discontinued due to intolerability to their assigned dose were then eligible for enrollment in an open-label extension study in which subjects could be titrated to and treated with arbaclofen at the best tolerated dose ranging from 5 mg BID to 15 mg TID (209FX303, NCT01555333). Results from the long-term open-label study will be reported separately.

Efficacy assessments were performed at baseline and treatment weeks 2, 4, and 8, as well as on phone calls to the primary caregiver at treatment weeks 1 and 3. Efficacy assessments on phone calls included only the CGI-I and CGI-S, which were obtained via an interview with the caregivers done by the site investigators. Safety and tolerability assessments were performed at baseline, treatment weeks 2, 4, 8 and the follow-up visit (which occurred 3 weeks after the end of treatment at week 8), as well as on all phone calls, and at 4 weeks after the follow-up visit for participants not entering the open-label extension. Families were queried about concomitant treatments and any changes in medications at every visit and phone call, in order to identify any emergent medical issues and ensure psychoactive medications were not being changed.

Study drug and matching placebo were provided as 5 and/or 10 mg orally disintegrating tablets in color-coded blister packs. The orally disintegrating formulation, which showed pharmacokinetics similar to racemic baclofen prior to use in 209FX301 and 209FX302, was developed for the studies to accommodate patients who could not swallow pills. Blinding was maintained in the setting of different doses by requiring subjects to all take the same number of tablets three times a day which could be either drug or placebo tablets. Subjects were assigned to treatment linked to a set of blister packs according to a centrally generated randomization list. Treatment compliance was monitored with a dosing form, which guardians completed on a daily basis.

### Assessments

#### Efficacy assessments

All efficacy outcomes were assessed as change from baseline after 8 weeks of treatment. The primary efficacy outcome for both studies was the parent or caregiver-rated Aberrant Behavior Checklist-Community Edition (ABC-C) refactored for FXS (ABC-C_FX_) Social Avoidance score. The key secondary outcome was the Clinical Global Impression-Improvement (CGI-I). The ABC-C_FX_ Social Avoidance score was chosen as primary endpoint because this measure showed improvement in the full intent-to-treat (ITT) cohort in the phase 2 study and because the FDA required a primary outcome in one behavioral domain. Because the drug reversed molecular, electrophysiological and synaptic phenotypes in the animal model [11, 17], it was postulated that it should help all aspects of FXS. Although the company requested, therefore, to nominate a measure of global function as the primary endpoint, this was not allowed by FDA. The FDA rather recommended that a global outcome measure be implemented as a key secondary rather than as primary endpoint; hence, the CGI-I was chosen as such key secondary measure. Furthermore, there was regulatory precedent for using the CGI-I (secondary) and the ABC-C Irritability subscale (primary), in addition to a responder analysis, for approval of atypical antipsychotics for irritable behavior in ASD [18]. Other secondary outcomes were the Clinical Global Impression-Severity (CGI-S), visual analog scale (VAS) for disruptive and anxiety behaviors, and Vineland Adaptive Behavior Scales, Second Edition (Vineland-II) - Socialization domain raw and standard scores (Survey Interview Form with parent/caregiver/Legally Authorized Representative (LAR)). Exploratory outcomes were a responder analysis (CGI-I score of 1 or 2, and 10, 20, 25, 30, 40, 50, and 60% improvement on the ABC-C_FX_ Social Avoidance subscale), the other ABC-C_FX_ Subscales (including Irritability, Hyperactivity, Stereotypic Behavior, Lethargy, and Abnormal Speech), Parenting Stress Index (PSI) – Short Form, Vineland-II Maladaptive Behavior Index and other domain raw scores, Vineland-II - Communication domain raw and standard scores, and Total Score and Daytime sleepiness subscale of the Children’s Sleep Habits Questionnaire (CSHQ). The ABC-C_FX_ was performed at baseline and weeks 4 and 8 of the treatment period. The CGI-I was performed at baseline and weeks 1, 2, 3, 4, and 8. The CGI-S and VAS were performed at baseline and weeks 2, 4, and 8. The Vineland-II, PSI, and CSHQ were performed at baseline and at 8 weeks.

#### Safety assessments

Safety and tolerability of STX209 was determined by adverse events (AEs; all visits and calls), physical examination, vital signs and weight (all visits), laboratory tests including complete blood count (CBC), chemistry panel, and urinalysis (UA) (baseline and weeks 4 and 8), electrocardiogram (ECG; baseline and week 8), and a suicidality assessment (three question interview with subject and parent/caregiver/LAR, all visits, as required by FDA guidelines). When the patient could not provide meaningful answers due to inadequate language or cognitive function, the family was asked if there was any sign of suicidality.

#### Pharmacokinetics

Four blood samples for analysis of plasma STX209 were obtained from each subject at four defined post-dose time points. Samples were to be used for population-based pharmacokinetic (PK) analyses, to confirm accurate randomization, and compliance with use of study medication. The goal was to perform an integrated population PK analyses with data from the phase 2 and 3 and open-label extension trials; however, these analyses were not completed prior to the wind-down of Seaside Therapeutics.

#### Description of assessments

##### DSM-IV-TR

Diagnostic and Statistical Manual of Mental Disorders IV – Text Revision (DSM-IV-TR) (APA 2000) criteria for Pervasive Developmental Disorders (PDD), including severe and pervasive impairment in several areas of development: reciprocal social interaction skills, communication skills, and the presence of stereotyped behavior, interests, and activities, were used by an Investigator on the study to determine at the screening visit if the subject had a PDD in addition to FXS.

##### ABC-C

The ABC-C is a 58-item parent-rated global behavior checklist implemented for the measurement of drug and other treatment effects in individuals with intellectual disability, and utilized in registration studies for drug efficacy in autism spectrum disorder [18]. In its original validation, five empirically derived dimensions were identified: Irritability, Lethargy/Social Withdrawal, Inappropriate Speech, Hyperactivity, and Stereotypic Behavior. The Lethargy/Social Withdrawal scale includes questions about social indifference, social avoidance, and physical lethargy [19]. A recent factor analysis of the ABC specifically in FXS (ABC-C_FX_) [16] generated a six-factor structure modifying items mapping to most subscales and identified a “Social Avoidance” factor that is related to the original “Lethargy/Social Withdrawal” scale, but which does not include the items assessing social indifference or physical lethargy. These items are now in a new subscale labeled Socially Unresponsive/Lethargic.

##### CGI-S

The CGI-S is a clinician-rated measure used to assess the impairment of neurobehavioral function in study subjects. The clinician should consider all aspects of that function, including but not limited to, internalizing and externalizing problems, and social engagement. The clinician’s score utilized the following 7-point Likert scale: normal (not at all impaired), borderline, mild, moderate, marked, severe, or extreme.

##### CGI-I

The CGI-I is a well-validated clinician-rated measure commonly used in drug studies [20] because it allows the clinician to integrate all sources of information, including the parent/caregiver history, observations in the clinic, and reports from other sources, into a single rating of improvement during treatment. For these studies, the investigator considered all aspects of the subject’s neurobehavioral function, including but not limited to internalizing problems, externalizing problems, and social engagement, and rated the scale employing a 7-point Likert scale: very much improved, much improved, minimally improved, no change, minimally worse, much worse, very much worse. In these studies, a single clinician-investigator at the site rated the CGI for each subject. This individual was not blinded to side effects or results of other measures, as it was not thought a priori that these would be unblinding based on the side effect profile from the phase 2 trial. Investigators were trained on CGI-I and CGI-S rating to standardize the rating of participants, including rating practice cases prior to performing the measure in the trials.

##### Vineland-II

The Vineland-II [21] is designed to assess the personal and social functioning of handicapped and non-handicapped persons. It is a gold standard for the assessment of adaptive functioning, and, with IQ testing, comprises one of two pillars for the assessment and diagnosis of intellectual disability. The “Survey Interview Form” of the Vineland-II was administered by a qualified psychologist or experienced rater to a parent or caregiver using a semi-structured interview format. Only the Communication domain, Socialization domain, and Maladaptive Behavior Index were completed (not the Daily Living Skills and Motor Skills domains).

##### VAS-Anxiety and Disruptive Behaviors

This methodology has been utilized in autism [18, 22]. The parent/caregiver/LAR is asked about the severity of anxiety and disruptive troublesome behaviors and is given examples of several of each of these types of target behaviors and then rates changes in severity of the anxiety and disruptive behavior target symptom on separate visual analog scales (VASs). The VAS is a 10-cm line, with troublesome behaviors anchored on one end with the description “worst ever” and on the other end with “no problem at all”. This scale showed good reliability in a prior study of subjects with FXS [23].

##### PSI – Short Form

The PSI [24] provides a measure of parental stress and is widely used in the assessment of family function for families with children who have special needs. The PSI was normed on over 2500 parents, and the 36-item short form provides a well-validated estimate of the overall stress faced by parents.

##### CSHQ

The CSHQ is a 35-item questionnaire designed for children aged 4 through 12 years, to screen for the most common sleep problems in that age group. Reliability and validity data has been collected on a sample of 495 elementary school children and on a clinical sample from a pediatric sleep clinic [25].

##### Suicidality assessment

This semi-structured interview of the parent/caregiver/LAR and subject was completed by a physician or clinical psychologist. A targeted set of questions were asked to assess potential suicidality. At a minimum, the clinician asked the subject the following questions: Do you ever wish you were dead? Have you done anything to hurt yourself? Then the clinician asked the parent/caregiver/LAR the following question: Has (subject’s name) done anything to hurt himself/herself (other than stereotyped self-injurious behaviors)?

#### Statistical analysis

For both studies, the ABC-C_FX_ Social Avoidance score was designated the primary endpoint based on the phase 2 study results [15]. All data collected in this study was documented using summary tables, figures, and subject data listings. All efficacy analyses were based on an intent-to-treat (ITT) population defined as all randomized subjects who were assigned to study medication, received at least one dose of double-blind study medication, and had post-baseline efficacy data available. For the primary efficacy variable of the ABC-C_FX_ Social Avoidance score, a per protocol (PP) population was also defined as those ITT subjects who fulfilled the entrance criteria and substantially adhered to the protocol for the duration of the study. Differences in efficacy variables from baseline to the end of 8 weeks of double-blind treatment in the arbaclofen treatment group and placebo treatment group were assessed using Restricted Maximum Likelihood (REML)-based Analysis of Covariance (ANCOVA) techniques for continuous variables and chi-square techniques for categorical variables as appropriate. Baseline scores and age were co-variates in the analyses. For all comparisons, a nominal *p* value of 0.05 or less was required to declare significance, and no adjustments for multiplicity were made. For the adolescent/adult study, there was one primary comparison of active versus placebo. For the child study, the level of significance for the primary efficacy comparison was protected using a closed testing procedure (that allows simultaneous testing of several hypotheses). In addition, only one key secondary efficacy variable was declared for each study.

The Safety Populations were comprised of all subjects who took at least one dose of study medication. Clinical safety was addressed by calculating the incidence of AEs in the two treatment groups and by descriptively summarizing laboratory assessments, physical examinations, ECG assessments, and vital signs.

#### Sample size

Study group sizes were based upon formal power analyses that identified the minimum number of subjects required to detect a medium effect size. Specifically, it did not appear smaller trials would be worth running as smaller group sizes would not be sufficiently powered to assess efficacy. For the primary outcome, the ABC-C_FX_ Social Avoidance subscale, the adolescent/adult study was designed to have at least 80% power to detect a treatment effect of size 0.55, with a *p* level of 0.05 in a sample size of (*n* = 60) subjects per group. The child study was designed to have at least 80% power to also detect a medium treatment effect of size 0.57, with a *p* level of 0.05 in a sample size of (*n* = 50) participants per group in each of the four dose arms.

## Results

### Demographic and background characteristics

**Fig. 2:**
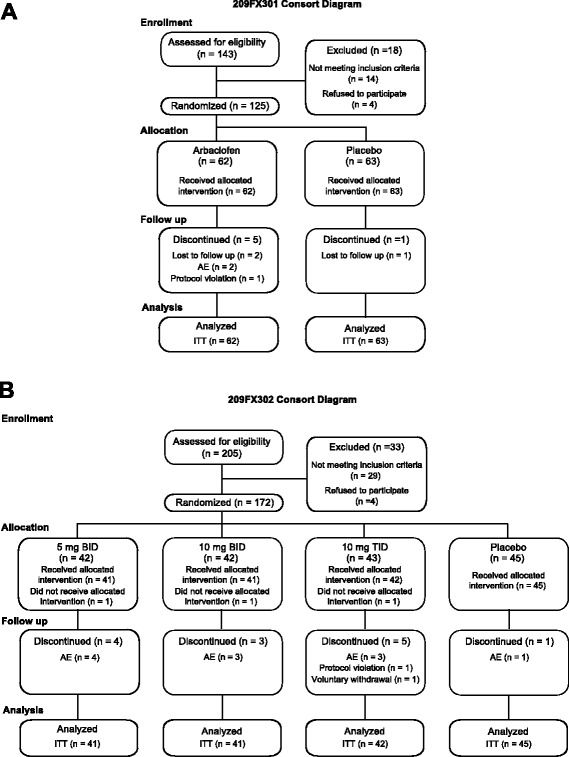
Consort diagrams for adult/adolescent (**a**) and child (**b**) phase III arbaclofen studies

A total of *n* = 125 adolescent/adult participants and *n* = 172 children with FXS were randomized into the two treatment arms (adolescents/adults) and four treatment arms (children) of each trial (Fig. 2, Tables 1 and 2). Enrollment in the child study had to be halted early for financial reasons and thus the projected sample size of 200 patients was not enrolled. Overall, *n* = 119 participants completed the adolescent/adult study and *n* = 159 completed the child study. A total of *n* = 13 subjects discontinued due to AEs, including two in the adolescent/adult study (both in the active treatment arm) and 11 in the child study (one in the placebo, four in the 5 mg BID, three in the 10 mg BID, and three in the 10 mg TID groups) (Fig. 2). The remaining discontinuations in the adolescent/adult study were due to protocol violation (one in treatment arm) and lost to follow-up (two treatment arm, one placebo), and in the child study were due to consent withdrawal or protocol violation (one each in 10 mg TID group). Protocol violations included dispensing the wrong kit for the adult/adolescent study subject and failure to meet inclusion/exclusion criteria in the child study subject.

**Table 1:** Demographics. Adult/adolescent study

Group/characteristic	Placebo (*N* = 63)	Arbaclofen (*N* = 62)	Total (*N* = 125)
Mean age (SD)	18.7 (7.22)	19.0 (6.73)	18.9 (6.96)
Age group (adolescents age 12–17)	36 (57.1%)	31 (50.0%)	67 (53.6%)
Gender (males)	49 (77.8%)	50 (80.6%)	99 (79.2%)
Race/ethnicity			
White non-hispanic	50 (79%)	47 (76%)	97 (77%)
White hispanic	4 (6.5%)	5 (8.1%)	9 (7.2%)
African American	6 (9.5%)	5 (8.1%)	11 (8.8%)
Asian	1 (1.6%)	1 (1.6%)	2 (1.6%)
Other	2 (3.2%)	4 (6.5%)	6 (4.8%)
Concomitant medications			
Any psychotropic medications	40 (63.5%)	35 (56.5%)	75 (60.0%)
Antipsychotics	11 (17.4%)	14 (22.6%)	25 (20.0%)
None	23 (36.5%)	27 (43.5%)	50 (40.0%)
DSM-IV diagnosis of ASD	45 (71.4%)	46 (74.2%)	91 (72.8%)
Mean ABC-C_FX_ Social Avoidance score (SD)	7.9 (3.48)	7.6 (3.17)

**Table 2:** Demographics. Child study

Group/characteristic	Placebo (*N* = 45)	Arbaclofen			Total (*N* = 169)
		5 mg BID (*N* = 41)	10 mg BID (*N* = 41)	10 mg TID (*N* = 42)	
Mean age (SD)	8.0 (2.20)	7.5 (1.86)	7.5 (1.81)	8.1 (2.02)	7.8 (1.99)
Gender (males)	38 (84.4%)	35 (83.3%)	35 (83.3%)	36 (83.7%)	144 (83.7%)
Race/ethnicity					
White non-hispanic	38 (84.4%)	29 (69.0%)	32 (76.2%)	36 (83.7%)	135 (78.5%)
White hispanic	2 (4.4%)	2 (4.8%)	4 (9.5%)	3 (7.0%)	11 (6.4%)
American Indian or Alaska Native	0	1 (2.4%)	0	0	1 (0.6%)
African American	2 (4.4%)	2 (4.8%)	1 (2.4%)	1 (2.3%)	6 (3.5%)
Asian	1 (2.2%)	3 (7.1%)	0	1 (2.3%)	5 (2.9%)
Other	2 (4.4%)	5 (11.9%)	5 (11.9%)	2 (4.7%)	14 (8.1%)
Concomitant medications					
Any	33 (73.3%)	34 (81.0%)	28 (66.7%)	32 (74.4%)	127 (73.8%)
Antipsychotics	12 (26.7%)	9 (21.4%)	4 (9.6%)	6 (14.0%)	31 (18.0%)
None	12 (26.7%)	8 (19.0%)	14 (33.3%)	11 (25.6%)	45 (26.2%)
DSM-IV diagnosis of ASD	34 (75.6%)	32 (76.2%)	35 (83.3%)	35 (81.4%)	136 (79.1%)
Mean ABC-C_FX_ Social Avoidance score (SD)	6.9 (3.49)	6.9 (2.84)	6.9 (3.03)	6.4 (2.92)	

*BID* twice daily, *TID* three times daily, *SD* standard deviation, *ASD* autism spectrum disorder, *ABC-C*_*FX*_ Aberrant Behavior Checklist refactored for FXS

In each study, participants’ demographic and background characteristics in the ITT population were generally comparable across the treatment groups (Tables 1 and 2). The mean age was about 19 years in the adolescent/adult study (range 12–48 years) and about 8 years (range 5–11 years) in the child study. In both trials, the majority of randomized patients were White non-Hispanic (77 and 78.5% in adolescent/adult and child studies, respectively). The higher proportion of males in both studies (79.2 and 83.7% in adolescent/adult and child studies, respectively) is a result of the reduced penetrance of FXS in females due to expression of the normal gene on the unaffected X chromosome. About 73% of adolescents/adults and 79% of children met clinical (DSM-IV) criteria for autism spectrum disorder (ASD). Baseline ABC-C_FX_ Social Avoidance scores were very similar across treatment groups, ranging from 7.6 to 7.9 in the adolescent/adult groups and from 6.4 to 6.9 in the child groups. Overall 20 and 18% of the adolescent/adult and child participants, respectively, were on antipsychotics, reflecting similar levels of usage in the two studies; however, there was some variability in antipsychotic use between the child groups, ranging from 9.6 to 26.7%, with the most antipsychotic use in the placebo group and the least in the 10 mg BID group (Tables 1 and 2).

### Adolescent/adult study efficacy results

The adolescent/adult study did not meet the primary objective of showing efficacy in reducing the ABC-C_FX_ Social Avoidance score after 8 weeks of treatment (Table 3) nor was there significant evidence of efficacy on any other measure including the CGI-I, CGI-S, VAS for disruptive and anxiety behaviors, Vineland-II - Socialization domain raw or standard scores, responder analysis, other ABC-C_FX_ subscales, PSI, Vineland-II Maladaptive Behavior Index, Vineland-II - Communication domain raw and standard scores, and Total CSHQ Score and Daytime sleepiness subscale. The CGI-S showed a trend toward improvement over placebo in the arbaclofen group (*p* = 0.063) but this was the only measure showing a strong trend, and given the multiple comparisons, this result alone is difficult to interpret as meaningful. The subgroup with co-morbid ASD also did not show benefit for arbaclofen over placebo.

**Table 3:** Efficacy measures at baseline and week 8 in ITT population for Adolescent/adult study

Measure	Placebo *N* = 63 (62 completers)			Arbaclofen *N* = 62 (57 completers)			
	Baseline	Week 8	Change	Baseline	Week 8	Change	*p*
ABC-C_FX_ SA	7.9 (3.48)	5.5 (3.37)	−2.4 (0.32)	7.6 (3.17)	5.3 (3.57)	−2.3 (0.33)	0.974
ABC-C_FX_ I	15.8 (13.18)	10.8 (11.01)	−5.3 (0.88)	16.9 (14.58)	12.3 (12.35)	−4.2 (0.89)	0.421
ABC-C_FX_ H	11.2 (7.32)	8.0 (6.75)	−3.4 (0.57)	12.9 (8.44)	8.7 (6.82)	−3.6 (0.58)	0.811
ABC-C_FX_ SB	8.4 (5.77)	6.0 (5.30)	−2.5 (0.40)	8.8 (5.37)	5.7 (4.84)	−2.9 (0.40)	0.508
ABC-C_FX_ L	12.3 (6.70)	7.3 (5.38)	−5.2 (0.56)	12.8 (7.19)	7.9 (5.94)	−4.7 (0.57)	0.536
ABC-C_FX_ IS	6.4 (3.74)	4.6 (3.02)	−1.8 (0.28)	6.5 (3.90)	4.8 (3.53)	−1.5 (0.29)	0.452
CGI-I	–	3.1 (0.12)	–	–	3.2 (0.12)	–	0.587
CGI-S	4.7 (0.92)	4.4 (0.98)	−0.3 (0.08)	4.6 (1.07)	4.1 (1.06)	−0.5 (0.08)	0.063
Responder	–	25.00%	–	–	24.10%	–	0.913
PSI	126.5 (20.45)	129.1 (23.26)	4.1 (2.23)	115.4 (22.04)	120.2 (22.48)	3.3 (2.27)	0.816
VAS-Anx	59.9 (24.57)	39.1 (26.82)	−21.2 (33.31)	62.0 (27.00)	46.4 (29.96)	−14.7 (3.38)	0.176
VAS-Dis	32.9 (28.75)	27.4 (24.74)	−7.0 (2.67)	37.6 (33.40)	33.3 (29.57)	−3.9 (2.72)	0.413
Vineland-II Soc	55.2 (20.33)	57.4 (19.70)	2.5 (1.17)	53.4 (15.80)	53.5 (18.17)	0.1 (1.17)	0.151
Vineland-II Comm	51.4 (20.91)	53.4 (20.56)	1.9 (1.06)	48.5 (18.54)	48.9 (19.72)	0.2 (1.06)	0.274
Vineland-II Mal	19.6 (1.43)	19.1 (1.27)	−0.6 (0.16)	20.0 (1.77)	19.2 (1.77)	−0.7 (0.16)	0.638
CSHQ-T	42.4 (6.68)	41.1 (5.83)	−1.3 (0.44)	42.7 (7.55)	41.1 (6.60)	−1.4 (0.45)	0.857
CSHQ-DS	10.8 (2.26)	10.7 (2.49)	−0.2 (0.25)	11.0 (2.83)	10.7 (2.49)	−0.3 (0.26)	0.700

Completers are those who finished the 8-week treatment period and assessments. All baseline, week 8 and change values given as mean (SE) for the group, except responder values which are given as percent responders out of total group, *p* values are for adjusted mean changes relative to the placebo group and adjusted mean changes are shown in the table

*ABC-C*_*FX*_ Aberrant Behavior Checklist refactored for FXS; *SA* Social Avoidance subscale, *I* Irritability subscale, *H* Hyperactivity subscale, *SB* Stereotypic Behavior subscale, *L* Socially Unresponsive/Lethargic subscale, *IS* Inappropriate Speech subscale, *CGI-I* Clinician Global Impression of Improvement, *CGI-S* Clinician Global Impression of Severity, *Responder* percent of participants with at least a 25% improvement on the ABC-C_FX_ Social Avoidance primary outcome and a CGI-I of 1 (very much improved) or 2 (much improved) at 8 weeks, *PSI* Parenting Stress Index, *VAS-Anx* Visual Analog Scale for Anxiety, *VAS – Dis* Visual Analog Scale for Disruptive Behaviors, *Vineland-II Soc* Vineland Adaptive Behavior Scales, Second Edition Socialization domain standard score, *Vineland-II Comm* Vineland Adaptive Behavior Scales, Second Edition Communication domain standard score, *Vineland-II Mal* Vineland Adaptive Behavior Scales, Second Edition – Maladaptive Behavior Index standard score, CSHQ-T = Children’s Sleep Habits Questionnaire – Total score, CSHQ-DS = Children’s Sleep Habits Questionnaire - Daytime Sleepiness subscale

### Child study efficacy results

**Fig. 3:**
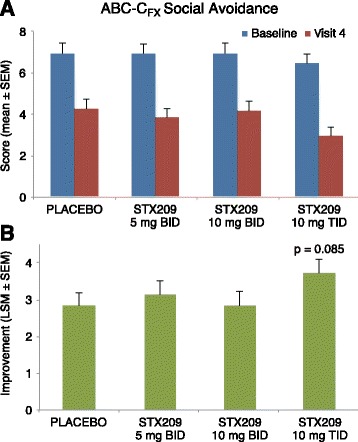
Baseline and end-of-treatment scores (**a**) and change in scores (**b**) for the primary outcome measure, the ABC-FX-Social Avoidance subscore, in child study for placebo and highest dose (10 mg TID) arbaclofen groups

**Fig. 4:**
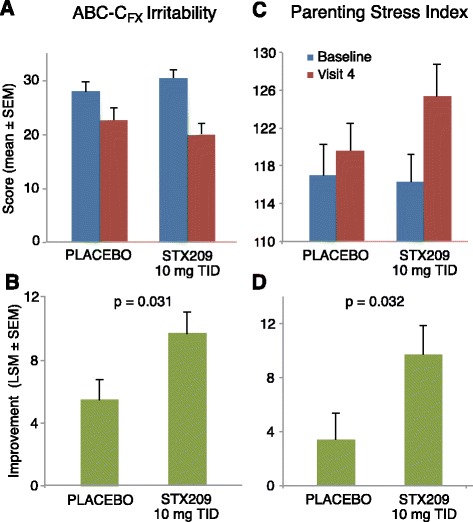
Baseline and end-of-treatment scores (**a**, **c**) and change in scores (**b**, **d**) for the ABC-FX-Irritability subscale (**a**, **b**) and Parenting Stress Index (**b**, **d**), in child study for placebo and highest dose (10 mg TID) arbaclofen groups. Standard error bars represent standard error of the mean

**Fig. 5:**
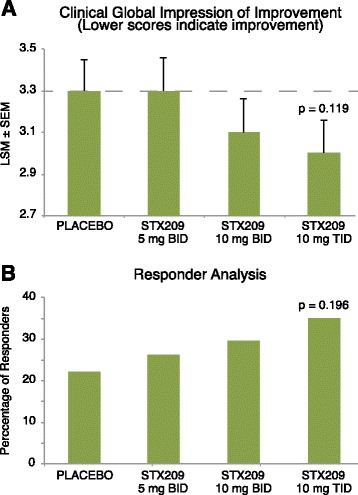
Dose responses for change in the CGI-I (**a**) and percent responders in responder analysis (**b**) in child study. Responders are defined as subjects with 25% improvement in the primary outcome ABC-C_FX_ Social Avoidance plus a CGI-I of 1 or 2

The child study did not meet the primary objective of showing efficacy in reducing the ABC-C_FX_ Social Avoidance score after 8 weeks of treatment (Table 4) in any dosing group. However, there was a strong trend toward improvement (Fig. 3) on this measure in the highest dose (10 mg TID) arbaclofen group over placebo (*p* = 0.085, effect size 0.24). This dose group (10 mg TID) at 8 weeks also showed nominally significant improvement (Fig. 4) for both the ABC-C_FX_ Irritability subscale (*p* = 0.031, effect size 0.51) and the Parenting Stress Index (*p* = 0.032, effect size 0.42), and trends toward improvement on the ABC-C_FX_ Hyperactivity subscale (*p* = 0.081, effect size 0.44) and the CGI-I (*p* = 0.119, effect size 0.43) (Fig. 5a). The Pearson Product Moment correlation coefficient for change during the 8 weeks of treatment for the ABC-C_FX_ Irritability subscale and PSI, the two measures with the highest significance for improvement on 10 mg TID arbaclofen, was 0.313 (*p* < 0.001). Substantial placebo effects were evident (Tables 4 and 5), and the lower dose groups did not show significant improvement for arbaclofen over placebo on any measure. Both the percent of predefined responders (those subjects with 25% improvement in the primary outcome ABC-C_FX_ Social Avoidance plus a CGI-I of 1 or 2) (Fig. 5b) and the CGI-I showed possible dose responses, with a numerically larger response as the dose increased although given the small cohort size these response trends did not achieve statistical significance. There were no significant effects in the highest dose group at 4 weeks, indicating effects were likely dependent on length of treatment. While taken together these trends and nominally significant results suggest a signal for drug effect, none of the effects were significant after correction for multiple comparisons.

**Table 4:** Efficacy measures at baseline and week 8 in ITT population for child study

Measure	Placebo *N* = 45 (44 completers)			Arbaclofen 5 mg BID *N* = 41 (38 completers)				Arbaclofen 10 mg BID *N* = 41 (39 completers)				Arbaclofen 10 mg TID *N* = 42 (38 completers)			
	Baseline	Week 8	Change	Baseline	Week 8	Change	*p*	Baseline	Week 8	Change	*p*	Baseline	Week 8	Change	*p*
ABC-C_FX_ SA	6.9 (3.49)	4.2 (3.24)	−2.8 (0.36)	6.9 (2.84)	3.8 (2.46)	−3.1 (0.38)	0.488	6.9 (3.03)	4.1 (2.85)	−2.8 (0.38)	0.968	6.4 (2.92)	2.9 (2.72)	−3.7 (0.38)	0.085
ABC-C_FX_ I	27.8 (12.91)	22.4 (14.53)	−5.5 (1.31)	29.7 (11.16)	21.7 (9.71)	−8.6 (1.41)	0.112	28.0 (10.81)	19.5 (11.81)	−8.8 (1.41)	0.112	30.4 (9.53)	21.6 (10.95)	−9.7 (1.41)	0.031
ABC-C_FX_ H	19.6 (7.82)	15.7 (8.28)	−4.0 (0.75)	21.6 (6.93)	17.2 (7.31)	−4.5 (0.81)	0.665	19.4 (6.58)	15.1 (7.57)	−4.6 (0.81)	0.577	21.9 (5.29)	15.9 (7.22)	−6.0 (0.81)	0.081
ABC-C_FX_ SB	9.3 (5.81)	6.3 (5.52)	−3.1 (0.50)	11.0 (4.81)	7.3 (4.26)	−3.5 (0.54)	0.613	9.6 (4.77)	6.7 (4.64)	−3.0 (0.54)	0.884	11.2 (5.01)	7.9 (5.12)	−3.2 (0.54)	0.908
ABC-C_FX_ L	11.6 (6.48)	7.3 (4.99)	−4.9 (0.64)	12.8 (6.05)	7.4 (5.03)	−5.4 (0.69)	0.577	13.3 (5.40)	8.0 (5.09)	−5.1 (0.69)	0.837	13.2 (5.08)	7.2 (5.94)	−5.8 (0.69)	0.324
ABC-C_FX_ IS	6.2 (3.86)	4.9 (3.76)	−1.5 (0.36)	7.2 (3.51)	5.3 (3.48)	−2.2 (0.38)	0.210	7.5 (3.52)	5.7 (3.93)	−1.6 (0.38)	0.868	7.9 (3.54)	6.1 (3.43)	−1.6 (0.38)	0.791
CGI-I	–	3.3 (1.00)	–	–	3.3 (1.16)	–	0.993	–	3.1 (0.96)	–	0.320	–	2.9 (0.87)	–	0.119
CGI-S	4.8 (0.79)	4.3 (1.01)	−0.4 (0.10)	5.1 (1.00)	4.7 (0.99)	−0.5 (0.11)	0.606	5.0 (0.89)	4.5 (0.91)	−0.4 (0.11)	0.700	4.9 (0.81)	4.4 (0.86)	−0.5 (0.11)	0.909
Responder	–	22.00%	–	–	26.30%	–	0.650	–	29.70%	–	0.432	–	35.10%	–	0.196
PSI	117.0 (21.97)	119.6 (19.65)	3.4 (1.96)	110.8 (17.46)	116.2 (15.34)	4.2 (2.18)	0.781	119.5 (20.07)	122.9 (18.83)	5.2 (2.11)	0.537	116.3 (18.97)	125.4 (20.93)	9.7 (2.16)	0.032
VAS-Anx	73.6 (18.99)	54.7 (25.54)	−19.1 (3.45)	75.0 (17.64	57.2 (24.05)	−18.8 (3.74)	0.957	77.7 (15.1)	55.8 (26.01)	−20.2 (3.71)	0.828	70.2 (16.37)	51.4 (27.43)	−18.8 (3.75)	0.950
VAS-Dis	58.0 (27.47)	47.2 (28.20)	−11.9 (3.31)	61.9 (25.83)	51.4 (25.59)	−10.6 (3.59)	0.787	61.5 (22.28)	46.6 (25.59)	−14.4 (3.54)	0.601	65.6 (23.69)	51.4 (27.10)	−10.7 (3.58	0.809
V-II Soc	64.0 (13.08)	66.0 (9.78)	3.1 (1.10)	64.8 (14.29)	68.9 (14.47)	3.6 (1.10)	0.191	67.5 (13.43)	69.3 (13.12)	1.9 (1.08)	0.866	64.5 (14.58)	66.7 (13.41)	3.1 (1.10)	0.314
V-II Comm	65.9 (12.92)	67.9 (10.79)	1.2 (0.72)	65.0 (12.51)	67.2 (10.78)	1.4 (0.80)	0.877	69.5 (14.81)	69.8 (14.89)	0.5 (0.78)	0.477	65.7 (11.88)	66.9 (11.88)	1.4 (0.79)	0.915
V-II Mal	20.2 (1.87)	19.5 (1.78)	−0.7 (0.18)	20.6 (1.31)	19.8 (1.42)	−0.7 (0.20)	0.877	19.8 (1.44)	19.3 (1.56)	0.6 (0.20)	0.667	20.5 (1.30)	19.4 (1.90)	−1.0 (0.20)	0.246
CSHQ-T	45.9 (7.98)	45.0 (7.41)	−1.5 (0.75)	47.3 (7.12)	46.3 (7.17)	−1.2 (0.76)	0.851	45.0 (6.97)	45.7 (7.60)	−0.1 (0.75)	0.402	47.2 (7.77)	45.9 (7.25)	−1.5 (0.75)	0.640
CSHQ-DS	11.1 (2.53)	11.1 (2.70)	−0.1 (0.28)	11.2 (2.83)	10.8 (2.43)	−0.5 (0.31)	0.310	11.2 (2.89)	10.9 (2.63)	−0.4 (0.30)	0.445	11.3 (2.70)	11.1 (2.12)	−0.3 (0.30)	0.619

Completers are those who finished the 8 week treatment period and assessments. All baseline, week 8 and change values given as mean (SE) for the group, except responder values which are given as percent responders out of total group, p values are for adjusted mean changes relative to the placebo group and adjusted mean changes are shown in the table

*BID* twice daily, *TID* three times daily, *ABC-C*_*FX*_ Aberrant Behavior Checklist-Community Edition refactored for FXS, *SA* Social Avoidance subscale, *I* Irritability subscale, *H* Hyperactivity subscale, *SB* Stereotypic Behavior subscale, *L* Socially Unresponsive/Lethargic subscale, *IS* Inappropriate Speech subscale, *CGI-I* Clinician Global Impression of Improvement, *CGI-S* Clinician Global Impression of Severity, *Responder* percent of participants with at least a 25% improvement on the ABC-C_FX_ Social Avoidance primary outcome and a CGI-I of 1 (very much improved) or 2 (much improved) at 8 weeks, *PSI* Parenting Stress Index, *VAS-Anx* visual analog scale for Anxiety, *VAS – Dis* visual analog scale for Disruptive Behaviors, *V-II Soc* Vineland Adaptive Behavior Scales, Second Edition – Socialization domain standard score, *V-II Comm* Vineland Adaptive Behavior Scales, Second Edition – Communication domain standard score, *V-II Mal* Vineland Adaptive Behavior Scales, Second Edition – Maladaptive Behavior Index standard score, *CSHQ-T* Children’s Sleep Habits Questionnaire – Total score, *CSHQ-DS* Children’s Sleep Habits Questionnaire - Daytime Sleepiness subscale

**Table 5:** Treatment-emergent adverse events (AEs) from baseline to week 8, by treatment (safety population)

	Adolescent/adult study		Child study			
Event	Placebo	Arbaclofen	Placebo	Arbaclofen 5 mg BID	Arbaclofen 10 mg BID	Arbaclofen 10 mg TID
	*n* (%)	*n* (%)	*n* (%)	*n* (%)	*n* (%)	*n* (%)
	*N* = 63	*N* = 62	*N* = 45	*N* = 42	*N* = 42	*N* = 43
Any adverse event	40 (63.5)	45 (72.6)	34 (75.6)	35 (83.3)	39 (92.9)	35 (81.4)
Vomiting	3 (4.8)	9 (14.5)	8 (17.8)	5 (11.9)	7 (16.7)	13 (30.2)
Aggression	1 (1.6)	2 (3.2)	8 (17.8)	0 (0.0)	5 (11.9)	7 (16.3)
Headache	6 (9.5)	9 (14.5)	3 (6.7)	1 (2.4)	2 (4.8)	5 (11.6)
Nausea	1 (1.6)	6 (9.7)	0 (0.0)	1 (2.4)	2 (4.8)	0 (0.0)
Diarrhea	3 (4.8)	5 (8.1)	3 (6.7)	5 (11.9)	5 (11.9)	5 (11.6)
URI	8 (12.7)	5 (8.1)	6 (13.3)	4 (9.5)	2 (4.8)	5 (11.6)
Rhinorrhea	2 (3.2)	1 (1.6)	2 (4.4)	2 (4.8)	2 (4.8)	4 (9.3)
Nasal congestion	0 (0.0)	2 (3.2)	2 (4.4)	3 (7.1)	2 (4.8)	3 (7.0)
Irritability	4 (6.3)	6 (9.7)	2 (4.4)	3 (7.1)	3 (7.1)	2 (4.7)
Agitation	2 (3.2)	5 (8.1)	3 (6.7)	3 (7.1)	3 (7.1)	2 (4.7)
Anxiety	1 (1.6)	5 (8.1)	1 (2.2)	4 (9.5)	6 (14.3)	3 (7.0)
Terminal insomnia	0 (0.0)	0 (0.0)	1 (2.2)	2 (4.8)	2 (4.8)	3 (7.0)
Middle insomnia	0 (0.0)	0 (0.0)	1 (2.2)	1 (2.4)	2 (4.8)	3 (7.0)
Decreased appetite	0 (0.0)	4 (6.5)	1 (2.2)	3 (7.1)	1 (2.4)	2 (4.7)
Ear infection	1 (1.6)	0 (0.0)	0 (0.0)	2 (4.8)	2 (4.8)	3 (7.0)
Gastroenteritis	0 (0.0)	2 (3.2)	0 (0.0)	2 (4.8)	1 (2.4)	3 (7.0)
Convulsion	1 (1.6)	0 (0.0)	0 (0.0)	0 (0.0)	0 (0.0)	0 (0.0)

*BID* twice daily, *TID* three times daily, *URI* upper respiratory infection

There was no improvement in any dose group for arbaclofen over placebo in the CGI-S, ABC-C_FX_ Socially Unresponsive/Lethargic, Stereotypic Behavior or Inappropriate Speech subscales, VAS for Anxiety, VAS for Disruptive Behaviors, Vineland-II - Socialization domain raw or standard scores, Vineland-II Maladaptive Behavior Index, Vineland-II - Communication domain raw and standard scores, and Total CSHQ Score and Daytime sleepiness subscale (Table 4). Analyses were performed by gender on the primary outcome for both the adult/adolescent and child studies and no significant effect of gender was found. In the child study, the cohort size was felt to be too small to analyze the subgroup with a co-morbid diagnosis of ASD for effects of arbaclofen in the highest dose group versus placebo.

### Safety results

The incidence of any treatment-emergent adverse events (AEs; Table 5) experienced by participants during the double-blind treatment period was similar in the arbaclofen and placebo groups (adults/adolescents: 40 (63.5% of participants) vs. 45 (72.6%), children on 10 mg TID dose: 34 (75.6%) vs. 35 (81.4%)). In children on the arbaclofen 5 mg BID and 10 mg BID, the incidence of any AEs was 35 (83.3%) and 39 (92.9%), respectively, reflecting lack of a dose relationship. Most reported AEs were mild in severity. AEs during the double-blind treatment period are shown in Table 5. In the adolescent/adult study, the most commonly experienced AEs were headache, nausea, vomiting, anorexia, irritability, anxiety, agitation, and upper respiratory infection (URI). All of these except URI were numerically more frequent in the arbaclofen group although no AE had an incidence of over 15%. In the child study the most commonly experienced AEs for the 10 mg TID, the highest dose group, were vomiting, aggression, URI, headache, rhinorrhea, nasal congestion, anxiety, insomnia, ear infection, and gastroenteritis. All of these except aggression, agitation, irritability, nausea, and URI were numerically more frequent in the highest dose arbaclofen group, although diarrhea, nasal congestion, anxiety, decreased appetite, and gastroenteritis were not dose related. Nausea, diarrhea, irritability, anxiety, insomnia, decreased appetite, ear infections, and gastroenteritis were all numerically slightly more frequent in the lower dose (5 and 10 mg BID) groups than in the placebo group (Table 5). Vomiting (30.3%) was the highest frequency side effect in the highest dose group, followed by aggression at 16.3% (placebo aggression incidence was 17.8%) and all other AEs had an incidence of less than 12%.

In the adolescent/adult study, there were two AEs leading to discontinuation, depressed mood, and psychotic disorder, both occurring in the arbaclofen group. In the child study there were 11 AEs leading to discontinuation (10 in an arbaclofen group and 1 in the placebo group). These were aggression/irritability (placebo group); anxiety, staring, and aggression (10 mg TID group); aggression, staring, and anxiety (10 mg BID group); and agitation (two participants), hyperactivity, and irritability (5 mg BID group). There were no clinically relevant changes in vital signs, weight, ECGs, or laboratory test results in any of the treatment groups in either study. There were no serious AEs or AEs related to suicidality in either trial and overall arbaclofen was well tolerated.

## Discussion

These trials represent the advanced stages of an effort at translating to humans with FXS the basic science and preclinical findings from the *Fmr1* knockout mouse, which suggested a potential therapeutic role of GABA-B agonists in ameliorating pathophysiology linked to excessive protein synthesis and/or diminished inhibition. The design of the studies was based on a phase 2 trial that had indicated benefits in social avoidance in patients with FXS treated with arbaclofen [15]. Unfortunately, neither the adolescent/adult nor the child study met the primary outcome.

### Interpretation of trial results

The groups enrolled in the studies seemed representative of the FXS population from a demographic standpoint. However, the rates of ASD were on the high end in comparison to previous studies [26, 27]. This is likely because the study enrolled based on social withdrawal, eliminating patients with better socialization. The male to female ratio was typical of most FXS studies performed in gender-mixed populations, with fewer females because they are less affected. Concomitant medication use was high overall but somewhat higher in the younger group, reflecting greater severity of behavior problems in the child study. Antipsychotic use was not particularly high in either group. However, as in most FXS cohorts, use was higher in the older population [28].

The adult/adolescent study did not show evidence of benefit for arbaclofen. However, the child study showed nominally significant improvement in several measures and dose-related trends for the primary and several secondary outcomes, suggesting a signal for a beneficial effect of the drug in the highest dose group. Specifically, nominally significant improvement was seen in the ABC-C_FX_ Irritability behavioral measure, which has been strongly linked to quality of life for FXS families in other studies [29]. A corresponding statistically correlated improvement in the parenting stress index (PSI) in the child study reported here is supportive evidence of potential benefit. This can be viewed as a potentially clinically meaningful result considering that the study involved only about 40 participants per group in placebo and comparison treatment groups. The primary outcome measure, ABC-C_FX_ Social Avoidance, showed a trend toward improvement that did not reach statistical significance. It is important to consider that the original power analysis estimated that 50 patients per group would be required to achieve statistical significance with a medium effect size. The study closed early for financial reasons and the groups were not fully enrolled. Although none of the significant effects identified would survive correction for multiple comparisons, the likelihood they are meaningful is increased considering the effect sizes and the fact that efficacy on the measures with the highest significance in the highest dose group tracked together. We suggest the trends for improvements observed on numerous outcome measures (ABC-C_FX_ Irritability, Social Avoidance and Hyperactivity subscales, the PSI and CGI-I) may represent therapeutic benefit of arbaclofen, particularly considering the variability in behavioral impairment in FXS and that there were no trends for worsening on any outcome measure.

It is noteworthy that the child study reported here is the first placebo-controlled trial conducted in FXS to date that has shown a nominally significant improvement in the ABC-C_FX_ Irritability or ABC-I (Irritability scale from original ABC-C), the primary efficacy endpoint for approval of antipsychotics in idiopathic ASD [18]. The failure to observe an improvement in the adolescent/adult study is possibly explained by the fact that the younger participants in the child study had a higher level of baseline irritability and, thus, it may have been easier to observe a response in this group. We also note that the average Vineland-II scores were higher in the children relative to the adolescents and adults. In a concurrent study of arbaclofen in idiopathic ASD, higher functioning individuals were found to have a greater drug response [30]. Alternatively or in addition, it is possible that disease modification with such a brief treatment occurs far more readily in the developing brain, prior to adolescence.

The drug effect sizes for multiple measures in the child study, including the ABC-C_FX_ Irritability, PSI, CGI-I, ABC-C_FX_ Social Avoidance, and ABC-C_FX_ Hyperactivity, between 0.24 and 0.51, were comparable or greater than the effect sizes observed in trials submitted to FDA for approved and marketed antidepressants (mean 0.31 [range 0.17–0.42]) [31]. In fact, to be able to detect a difference at 80% power with 38 subjects per group on the primary outcome, the ABC-C_FX_ Social Avoidance subscale, an effect size of 0.65 would be needed (*n* = 40, effect size 0.63), a higher effect size than seen for most marketed psychotropic drugs. Effect sizes for risperidone [18] in ASD were higher on the ABC-C Irritability subscale at 1.2, although only 0.4 for the ABC-C Social Withdrawal subscale. However, while the risperidone study was conducted in the absence of concomitant medication for co-morbid behavioral diagnoses, in this study arbaclofen achieved the aforementioned effect sizes over and above treatment with optimized concomitant medications to manage behavior, and in about a quarter of cases, in addition to therapy with risperidone or aripiprazole. The allowance of concomitant medication used for approved indications to treat co-morbid diagnoses in FXS is expected to confound and reduce observable efficacy of arbaclofen, yet allows identification of effects contributed by arbaclofen which supersede those obtained from standard care. Furthermore, the scientific rationale for development of arbaclofen was based upon the ability to correct the underlying molecular perturbations, not a specific behavior, and the conceptual treatment target was really the full FXS phenotype. The trial results are consistent with this concept in that beneficial effects were distributed across multiple behaviors and effect sizes and increased with longer durations of treatment. Additionally, the apparent dose dependence of numerical improvement on ABC-C_FX_ Irritability, ABC-C_FX_ Social Avoidance, CGI-I, and the responder analysis suggest that higher doses have the potential to provide even greater benefit. The excellent tolerability observed with all doses in both studies, and the lack of a substantially higher frequency of adverse events or study discontinuation at higher doses in the child study, suggest it would be safe to explore efficacy at higher doses in future studies of arbaclofen in individuals with FXS. However, dropouts for side effects may have been dose-dependent in the child study. Thus, the lack of an option to down-titrate for dose adjustment in this study was another limitation that may have reduced the ability to detect beneficial effects of arbaclofen.

### Outcomes measurement as a challenge to translation

A potential limitation related to clinician CGI ratings and thus responder analyses in this study is the lack of blinding of CGI raters to adverse events. However, as seen in Table 5, side effects were not sufficiently more common in the arbaclofen groups over placebo to unblind the investigators. Although raters underwent training to rate individuals with FXS on the CGI-S and CGI-I, a further limitation of the clinician CGI ratings in these studies was the lack of formal anchors and inter-rater reliability requirements for raters before performing ratings in the trials.

The ABC-C_FX_ scale may be adequate for detecting behavioral effects, as seen for the ABC-C_FX_ Irritability scale in the arbaclofen child study. Nonetheless, there are problems with these scales, particularly if the same scale is used both to qualify for study participation and as a primary outcome measure. Families may exaggerate symptoms in the domain required to get into the trial and then “regression to the mean” leads to large placebo effects during the trial. This may partially explain why in the previous phase 2 arbaclofen study, in which enrollment was based on ABC-C Irritability, subjects showed improvement over placebo in ABC-C_FX_ Social Avoidance but not Irritability [15]. The large placebo effect on Irritability in that study may have rendered a drug effect impossible to detect. Conversely, enrollment in the phase 3 child study here was based on ABC-C_FX_ Social Avoidance, and the improvement on arbaclofen relative to placebo was greater for the Irritability than for the Social Avoidance subscale. This experience illustrates the potential for high placebo response rates when utilizing parent-rated outcome measures. There is a clear need to explore whether alternative enrollment strategies can reduce placebo response rates, to develop and validate alternative outcome measures, and to use study designs that address exaggerated enrollment scores (e.g., placebo lead-in).

In general, placebo effects in typically developing children and in neurodevelopmental disorders are large and may consequently mask results. They may also be correlated with severity of externalizing problems (e.g., aggression) and parental expectations. Clinician-ratings are potentially less susceptible than parent-ratings to these issues; thus, it may be better to use clinician-anchored scales when possible, although truly objective measures would presumably be even better and are badly needed for future FXS trials, especially in areas like language, cognition, daily living skills, and social function (also discussed in Budimirovic et al., this issue [32]). As a result of the recognition of placebo response and variability problems with parent-rated measures in these and other early large trials in FXS, subsequent trials have moved away from use of parent-rated forms and have incorporated a larger emphasis on clinician-rated measures including the Vineland-II [33], the Pediatric Anxiety Rating Scale [34], and a Fragile X Syndrome Rating Scale [35]. Ongoing validation studies are underway for measures involving direct observation or testing in FXS, such as expressive language sampling [36] and the NIH Toolbox cognitive battery [37], and these types of measures have been increasingly incorporated in subsequent trials, including the KiTAP computerized executive battery [38] and expressive language sampling. In addition, objective biomarkers to demonstrate target engagement such as eye tracking [39, 40] and event-related potentials are being incorporated into proof-of-concept trials.

The results of the child study may suggest a certain specificity of arbaclofen effects for irritability in FXS. However, there are alternative explanations related to the attributes of the ABC-C_FX_ and other outcome measures employed in these studies. Parents seem to be more reliable reporters of severely irritable/aggressive behavior than other types of behavior (e.g., social). Furthermore, the FXS-specific factor analysis of the ABC-C led to a new subscale, the ABC-C_FX_ Social Avoidance subscale. Nevertheless, the latter was reduced to only four questions (i.e., Seeks isolation, Withdrawn, Isolates self, Prefers to be alone). While the validity of the ABC-C_FX_ Social Avoidance subscale is supported by a previous observational study categorizing social withdrawal behaviors in FXS [41], the limited number of behaviors in the ABC-C_FX_ Social Avoidance is likely to reduce its sensitivity for detecting improvements over time.

It is possible that in the reported FXS trials multiple domains improved in responders, but with variable magnitude reflecting the individual variability in severity of core and associated features in FXS. It would be optimal to be able to measure the drug effect across multiple domains to capture this potentially variable response. Indeed, an “autopsy” of the arbaclofen phase 3 FXS trials, focused on differentiating drug and placebo response characteristics, suggested that improvements on arbaclofen were not fully reflected by the outcome measures utilized in the trials [42]. Specifically, it appears that improvements in several domains were not captured and future trials may benefit by including assessments of language and cognition. In this informal analysis, improvement in the ability to avoid a maladaptive response to a situation perceived by the FXS child as overwhelming or aversive (families often referred to this as “coping”) was the response characteristic most enriched in the arbaclofen responses relative to placebo responses. One can imagine a construct in which such maladaptive responses may result in aggression, social avoidance and/or hyperactivity in different individuals for different situations. Consequently, if arbaclofen increased the ability to deal adaptively with, or decreased the perception of aversiveness of, previously challenging settings and situations, improvement in several behavioral domains would be expected as a part of a single therapeutic effect. Thus, diversity of symptoms and drug benefits that map into different domains in different situations and patients complicate assessments in FXS trials. Obviously, animal studies based on improvements in synaptic function provide little guidance on the best measures of improvement in humans. Therefore, it will be important to develop methods for measuring and analyzing drug response that will cover multiple affected domains in humans.

### New designs and paradigms to assess validity of effects in animal models

The relatively weak statistical trends seen only in the child trial raises the possibility that the etiologic model could be wrong and that mGluR5- and GABA-related abnormalities correctable in the animal model do not translate to humans. However, the trials performed to date do not provide definitive answers, as lack of efficacy could also result from a variety of factors affecting the outcome of the studies. As discussed above, these include inadequate trial design for translation of results from animal models to man, insensitive outcome measures, a need to measure outcomes across the spectrum of core and associated phenotypes rather than a single behavior, need to test the targeted treatments in younger patients with FXS due to the developmental nature of the condition and lack of clarity about the duration of plasticity windows, need for optimizing individual dosage and longer exposure times to see change in a developmental condition, and need to sort out response variability with biomarkers both to identify potential responders and to establish target engagement.

Further, effect sizes in the child study for numerous measures in the high dose group are comparable to those of marketed psychotropic drugs and for phenotype reversal in animal models. These results suggest that lack of statistical significance is due to enrollment of a cohort size that was underpowered for significance. In comparison, definitive trials for registration of commonly used antidepressants in adults typically enrolled between 300 and 1100 subjects [31] to achieve statistical significance for an effect size of 0.31. A meta-analysis of all published antidepressant trials performed in children and adolescents (*n* = 3000) identified comparable or slightly smaller effect sizes in children and adolescents [43]. Statistical significance is achieved in these trials by enrolling larger cohorts than are practical for rare disorders such as FXS. We suggest that clinicians consider effect size, number needed to treat, or success rate differences [44], in addition to *p* values, when assessing benefit of novel therapeutics. Furthermore, we suggest that multi-component outcome analyses at the group and subject level, using strategies such as permutation testing [45], are likely to better index the overall effect size in rare genetic disorders by taking into account the entire condition and may be a helpful strategy for assessing efficacy in smaller cohorts.

A larger study designed to address all the above issues will be required to assess whether or not the etiologic model is wrong. Indeed, a flexible dose trial enrolling larger cohorts of children titrated to the optimal dose of drug or placebo, employing a multi-component primary outcome that evaluates FXS-related problems across behavioral, social, and language phenotypes and also incorporates parenting stress and quality of life, would represent a more thorough evaluation of arbaclofen efficacy in FXS. In a genetically defined developmental condition with variable impairment across multiple cognitive and behavioral domains, it is likely that key outcome definitions will need to address the entire condition rather than one behavior, for successful translation to humans of targeted treatments that correct synaptic phenotypes in animal models.

## Conclusions

FXS has been a model neurodevelopmental disorder for clinical trials directed at translating to humans the treatments that correct core pathophysiological mechanisms in animal models. It seems clear from the trials reported here and in other trials reported to date [46] that such translation to humans is challenging and that it will be important to learn from these early attempts to improve methodology and the drug development process. Despite failing to document significant improvement on the prospectively defined primary endpoints, arbaclofen appears to provide therapeutic benefit in some individuals with FXS. These encouraging results suggest arbaclofen should be studied further to replicate the result. Young age, higher doses, larger cohort sizes, trial designs that minimize placebo effect, and better outcome measures covering a wide range of potential responses are among the factors that may allow success in future trials of arbaclofen and other drugs that have shown promise in FXS experimental models.
